# Gliovascular and cytokine interactions modulate brain endothelial barrier *in vitro*

**DOI:** 10.1186/1742-2094-8-162

**Published:** 2011-11-23

**Authors:** Ganta V Chaitanya, Walter E Cromer, Shannon R Wells, Merilyn H Jennings, P Olivier Couraud, Ignacio A Romero, Babette Weksler, Anat Erdreich-Epstein, J Michael Mathis, Alireza Minagar, J Steven Alexander

**Affiliations:** 1 Department of Molecular and Cellular Physiology, School of Graduate Studies, Louisiana State University Health Sciences Center-Shreveport, 1501 Kings Hwy, Shreveport, LA 71130, USA; 2 Cell Biology and Anatomy, School of Graduate Studies, Louisiana State University Health Sciences Center-Shreveport, 1501 Kings Hwy, Shreveport, LA 71130, USA; 3 Department of Neurology, School of Medicine, Louisiana State University Health Sciences Center-Shreveport, 1501 Kings Hwy, Shreveport, LA 71130, USA; 4 Inserm, U1016, Institut Cochin, Paris, France; 5 Cnrs, UMR8104, Paris, France; 6 Univ Paris Descartes, Paris, France; 7 Department of Biological Sciences, The Open University, Milton Keynes, UK; 8 Department of Medicine, Weill Medical College, 1300 York Ave, New York, NY-10065, USA; 9 Division of Hematology-Oncology, Departments of Pediatrics and Pathology, The Saban Research Institute at Children's Hospital Los Angeles and Keck School of Medicine, University of Southern California, 4650 Sunset Boulevard, Los Angeles, California 90027, USA

**Keywords:** TNF-α, IL-1β, IFN-γ, Brain endothelium, Astrocytes, Co-culture, Mono-Culture

## Abstract

The glio-vascular unit (G-unit) plays a prominent role in maintaining homeostasis of the blood-brain barrier (BBB) and disturbances in cells forming this unit may seriously dysregulate BBB. The direct and indirect effects of cytokines on cellular components of the BBB are not yet unclear. The present study compares the effects of cytokines and cytokine-treated astrocytes on brain endothelial barrier. 3-dimensional transwell co-cultures of brain endothelium and related-barrier forming cells with astrocytes were used to investigate gliovascular barrier responses to cytokines during pathological stresses. Gliovascular barrier was measured using trans-endothelial electrical resistance (TEER), a sensitive index of *in vitro *barrier integrity. We found that neither TNF-α, IL-1β or IFN-γ directly reduced barrier in human or mouse brain endothelial cells or ECV-304 barrier (independent of cell viability/metabolism), but found that astrocyte exposure to cytokines in co-culture significantly reduced endothelial (and ECV-304) barrier. These results indicate that the barrier established by human and mouse brain endothelial cells (and other cells) may respond positively to cytokines alone, but that during pathological conditions, cytokines dysregulate the barrier forming cells indirectly through astrocyte activation involving reorganization of junctions, matrix, focal adhesion or release of barrier modulating factors (e.g. oxidants, MMPs).

## Background

The blood brain barrier (BBB) is a unique astrocyte-capillary-endothelial complex which maintains CNS homeostatic fluid balance, and serves as a first line of defense protecting the brain and parenchyma against pathogens, as well as blood-borne leukocytes and hormones, neurotransmitters and pro-inflammatory cytokines and chemokines [1,2]. The loss of BBB structural integrity and function plays a central role in the pathogenesis of neuroinflammatory diseases like multiple sclerosis, Alzheimer's disease, meningitis, brain tumors, intracerebral hemorrhage and stroke [3-10]. Many reports in the literature indicate that loss of BBB in neuroinflammation represents a result of complex often continuous interactions between the BBB and immune cells, adhesive determinants and inflammatory cytokines, all of which may be relevant targets for therapy [11-18]. While several studies have modeled interactions between astrocytes and brain endothelial cells, fewer studies have considered how this gliovascular unit might be dysregulated by the combined influences of metabolic stress and cytokine exposure.

Astrocytes are the most abundant glial cells in the CNS, playing crucial roles in cerebral ion homeostasis, neuro-transmitter regulation, structural and metabolic support of neuronal and endothelial cells and BBB maintenance [19-21]. Furthermore, astrocytes provide an important link between neuronal and vascular units in the glucose-lactate shuttle and in modulating Ca^2+ ^responses [22-29]. Importantly, astrocytes have been shown to play divergent roles in various pathologic conditions [29-32]. For example, following ischemic strokes, astrocytes protect neurons [33-35] by secreting several neurotrophic factors like glial cell-line derived neurotrophic factor [36], neurotrophin-3 [37,38], transforming growth factor-β1 [39], and vascular endothelial growth factor [40]. Astrocytes can also secrete pro-inflammatory cytokines such as TNF-α, IL-1β, and IL-6 which would be anticipated to aggravate inflammatory injury to ischemic tissues [41]. The roles played by astrocytes and astrocyte-derived factors in maintaining or injuring the post-ischemic BBB are complex, cell-specific and time-dependent. Several reports have indicate that astrocytes co-cultured with endothelial cells or astrocyte-conditioned media improve endothelial barrier integrity, however the potential effects of astrocytes on the cerebral endothelial cells during CNS stress contributing to the pathological loss of BBB are not yet as well understood [20]. The mechanisms through which factors secreted by stressed astrocytes (e.g. in response to glucose, serum, or oxygen deprivation) dysregulate endothelial barrier during pathologies e.g. cerebral ischemia remains an area under intensive investigation [42].

Cytokines exert diverse and cell-specific effects on BBB integrity [43-46]. TNF-α and IFN-γ are among the best studied cytokines which cause differing permeability responses in different cell systems [47]. For example, IFN-γ was shown to increase permeability in human colonic epithelial cells (T84), microvascular endothelial cells, human umbilical vein endothelial cells and cholangiocytes, but decreased permeability in human lung epithelial cells (Calu-3). TNF-α increases permeability of bovine pulmonary artery endothelial (BPAEC) monolayers, human colonic adenocarcinoma (Caco-2), HT29/B6 and cholangiocytes, but decreased solute permeability of uterine epithelial cells (UECs) [47]. Further, TNF-α can either increase or decrease solute exchange depending on the type of insult in porcine renal epithelial cells (LLC-PK1) [48,49]. These effects are mediated by diverse mechanisms involving actin reorganization, monolayer motility, NF-kβ activation, apoptosis and reorganization of junctional proteins [49-54].

Apart from direct actions of cytokines, factors secreted by astrocytes may also disturb BBB [32,42]. For example, matrix metalloproteinases ('MMP') -9 (MMP-9) and -13 (MMP-13), derived in part from astrocytes may contribute to post-ischemic BBB dysregulation [55-57] and MMP-9 inhibition partially protects against ischemic stroke, decreasing infarct size and BBB breakdown. Conversely, Tang et al. have reported that MMP-9^-/- ^mice exhibit a more pronounced BBB damage and edema than controls (in a collagenase model of hemorrhage) [58]. Many other mediators may be involved in mediating the deleterious effect of stressed astrocytes on BBB during pathological conditions.

In the present study we investigated the direct or indirect influence of cytokines (TNF-α, IL-1β and IFN-γ) on brain endothelium and astrocytes (individually or in synergy) on barrier during metabolic stresses using a 3-D *in vitro *BBB model with human, mouse brain endothelial cells, ECV-304 and astrocytes. The results of our current study indicate that under conditions of pathological stress, astrocytes indirectly modify endothelial barrier responses to cytokines, leading to strikingly different barrier conditions observed in the absence of astrocytes. The differential roles of astrocytes and cytokines in modulating brain endothelial barrier properties are also discussed.

## Materials and methods

### Reagents

Mouse rTNF-α, was purchased from Endogen (Woburn, MA) Thermo scientific (Rockford, IL), Mouse rIL-1β was purchased from Chemicon (Temecula, CA) or Endogen. Mouse rIFN-γ was purchased from Endogen. Human rTNF-α and rIFN-γ were purchased from Thermo-scientific. Human rIL-1β was purchased from Endogen. All other chemicals were purchased from Sigma (St. Louis, MO) unless specified.

### Cell culture

Murine brain endothelial cells (bEnd.3) provided by Dr. Eugene Butcher (Stanford Univ.). Human fetal astrocytes (HFA) were provided by Dr. Danica Stanimirovic (Univ. of Ottawa). Both cell types were both cultured in DMEM supplemented with 10% fetal calf serum (Hyclone) and 1% Penicillin-Streptomycin-Amphotericin (PSA) ('complete medium' referred as 10% DMEM). Media were changed every 2^nd ^day. Human brain endothelial cell line (HBMEC-3) was kindly provided by Dr. Anat Erdreich-Epstein, (Children's Hospital of Los Angeles, California) and were cultured in RPMI with 10% FCS with 2 mM sodium pyruvate and 1% PSA. An additional human brain endothelial cell line (HCMEC-D3) was provided by Dr. P.O. Couraud, (Institut Cochin, Paris, France) [59,60]. HCMEC-D3 cells were cultured in rat tail collagen coated plates (100 ug/ml) in medium consisting of EBM2 supplemented with 5% FCS, 1.4 uM hydrocortisone, 10 mM HEPES, 1 ng/ml bFGF and 1% PSA. As an additional control, ECV-304, (ATCC, Manassas, VA) a bladder carcinoma with several endothelial-like properties was also used in this study [61]; (these cells were cultured as described for HBMEC-3.)

### In vitro barrier function studies

Brain endothelium (and ECV-304) was cultured on the apical surface of 8.0 μm PETP transwell inserts (Falcon) placed in a 24-well culture plates ('outer chamber'). The outer chamber contained 1 ml of medium with 0.5 ml media in the insert. To generate contact-independent co-cultures, the apical/inner surface of the insert was seeded with either human or mouse brain endothelial cells or ECV-304 cells; astrocytes were cultured in the basal/outer chamber.

To create a 'close-contact' co-culture system closely resembling the *in vivo *gliovascular unit, after human or mouse endothelial (HCMEC-D3 or bEnd-3) cells were cultured on the apical surface and astrocytes were cultured on the basal side of the insert. These cultures were established by allowing 100 μl of astrocyte cell suspension (approximately 20,000 cells) to adhere to the basal surface for 1 hr before seeding the apical surface of the insert with endothelial cells. Later, inserts with attached endothelial cells and astrocytes were transferred into the outer chamber.

### Trans-endothelial electrical resistance (TEER)

Trans-endothelial electrical resistance was measured using an epithelial volt-ohmmeter (EVOM) (World precision instruments, Sarasota, FL). Cultures systems on inserts were exposed to treatments, and at time points, were transferred to the TEER chamber (using matching media conditions) and electrical resistance recorded (ohms/cm^2^, no = ohms/0.33^2^).

### Brain endothelial barrier permeability

Mouse brain endothelial cells (bEnd3) were grown in transwell inserts (apical side) and at confluence were treated with cytokines in both apical and basal sides. TEER was recorded at 24 h time intervals. At 3 d, 50 μl of FICT-dextran (120 kD) at a final concentration of 1 mg/ml (in culture medium) was added to the apical side of the brain endothelium. At various time points from 30 min to 6 h, 100 μl of medium from the basal chamber was used to measure the extravasated FITC-dextran to the basal side across the endothelium. Equal volume of media was supplemented to replace the volume of used medium. The experiment was terminated after 6 h. All the readings were measured at constant 'gain' settings. The values obtained were plotted on graph pad and checked for significance.

### Cytokine treatments

Murine brain endothelial cells and human astrocytes were treated with matching mouse or human TNF-α (20 ng/ml), IL-1β (20 ng/ml) and IFN-γ (1000 U/ml) respectively. Depending on the study, cytokines (at specified concentrations) were added either to the apical or basal surface surrounding the insert (in contact-dependent or contact-independent systems).

### MTT assay

Brain endothelial cells were grown in 96-well plates. At confluence, human and mouse brain endothelium was incubated with matching TNF-α (20 ng/ml), IL-1β (20 ng/ml), IFN-γ (1000 U/ml) for 4 d. At the end of incubation time period, cell energy metabolism was measured by washing cells 3X, and extracting in 300 ul of acetic acid/isopropanol. Absorbance of the acid/isopropanol-extracted products was then measured at 450 nm.

### Statistics

Graphpad-3 InStat™ software was used to perform statistical analyses. One way-ANOVA or repeated measures ANOVA each with Dunnett's' post-hoc test or Bonferroni post-test were used to determine statistical significance. Sigmaplot™ was used to generate plots. *p < 0.05 was considered to be statistically significant, **p < 0.01 very significant, and ***p < 0.001 highly significant.

## Results

### 1a. Effect of mouse cytokines (apical + basal exposure) on mouse brain endothelial barrier (mono-cultures)

#### Control

Under control (untreated) conditions, barrier gradually diminishes over 7 days to 47.8 ± 1.2% of baseline. Control cultures' barrier at 0 d was 276.67 ± 14.98 ohms/cm^2 ^and at 7 d was 140.17 ± 3.97 ohms/cm^2^.

#### TNF-α

There was a slight decrease in mouse brain endothelial barrier treated with TNF-α till day 7. This reflects a cumulative treatment on both apical + basal sides. No difference was observed in the mouse brain endothelial barrier treated either apically or basally. At day 7 the barrier was still higher than controls (81.72 ± 1.6 vs. 47.8 ± 1.2% of baseline). TNF-α treated cultures barrier at 0 d = 274.67 ± 6.0 ohms/cm^2 ^and at 7 d = 224.17 ± 1.5 ohms/cm^2^.

#### IL-1β

A gradual decrease in mouse brain endothelial barrier was observed in cells treated with IL-1β through day 7. However, at day 7 the barrier was still slightly higher than controls (60.3 ± 2.2 vs. 47.8 ± 1.2% of baseline). At 0 d, IL-1β treated cultures resistance was 269.83 ± 3.83 ohms/cm^2 ^and at 7 d = 162.83 ± 4.09 ohms/cm^2^.

#### IFN-γ

**Figure 1:**
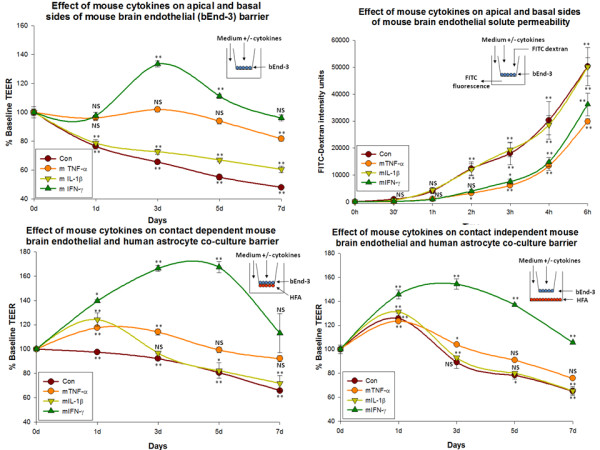
**Effect of mouse cytokines on bend-3 mono-culture barrier and bEnd-3/HFA co-culture barrier**. a) Cumulative effect of mouse cytokines (TNF-α (20 ng/ml), IL-1β (20 ng/ml) and IFN-γ (1000 U/ml)) applied to apical + basal sides of mouse brain endothelial mono-cultures. Resistance was recorded daily (7 d). Significant increase in the resistance of mouse brain endothelium was observed in a rank order of IFN-γ > TNF-α > IL-1β compared with control. Inset shows the mode of culture and cytokine treatment. Bars indicate standard error. Repeated measured ANOVA with Dunnett's post-hoc test. *p < 0.05 was considered to be statistically significant, **p < 0.01 very significant, and ***p < 0.001 highly significant. b) Effect of mouse cytokines (TNF-α (20 ng/ml), IL-1β (20 ng/ml) and IFN-γ (1000 U/ml)) of mouse brain endothelial solute permeability. Solute permeability was measured at 30', 1 h, 2 h, 3 h, 4 h and 6 h after 3 d of treatment. TNF-α and IFN-γ treated cultures showed lesser permeability than control or IL-1b treated cultures. The solute permeability of mouse brain endothelium in this experiment was in a rank order of IFN-γ ≈ TNF-α > IL-1β ≈ Con. Bars indicate standard error. Repeated measured ANOVA with Dunnett's post-hoc test. *p < 0.05 was considered to be statistically significant, **p < 0.01 very significant, and ***p < 0.001 highly significant. c) Effect of mouse cytokines (TNF-α (20 ng/ml), IL-1β (20 ng/ml) and IFN-γ (1000 U/ml)) on contact dependent bEnd-3/HFA co-culture system. Resistance was recorded daily. Significant increase in mouse brain endothelial barrier was observed with IFN-γ > IL-1β ≥ TNF-α compared to controls. Inset shows the mode of contact dependent system used and cytokine addition. Bars indicate standard error. Repeated measures ANOVA with Dunnett's post-hoc test. d) Effect of mouse cytokines (TNF-α (20 ng/ml), IL-1β (20 ng/ml) and IFN-γ (1000 U/ml)) on contact independent bEnd-3/HFA co-culture system. Resistance was recorded daily. Significant increase in the resistance of brain endothelium was observed with IFN-γ > IL-1β ≥ TNF-α compared with control. Inset shows the mode of contact dependent system used and cytokine addition. Bars indicate standard error. Repeated measures ANOVA with Dunnett's post-hoc test. *p < 0.05 was considered to be statistically significant, **p < 0.01 very significant, and ***p < 0.001 highly significant.

We observed an increase in mouse brain endothelial barrier with IFN-γ over the other 2 cytokines or controls at all time points. The maximal resistance of brain endothelium treated with IFN-γ was reached at day 3 (133.5 ± 2.1% of baseline). The resistance decreased from day 3, but remained still higher than untreated controls at day 7 (96.0 ± 2% vs. 47.8 ± 1.2%) (Figure 1a). Resistance of cultures treated with IFN-γ at 0 d = 261.67 ± 3.2 ohms/cm^2 ^and at 7 d = 251.33 ± 6.7 ohms/cm^2^. The rank order of TEER in this experimental model was IFN-γ>TNF-α>IL-1β>Con. Inset shows the mode of culture and treatment.

### 1b. Effect of mouse cytokines (apical and basal) on brain endothelial barrier (monoculture) solute permeability

Solute permeability measurements using FITC-dextran extravasation across endothelial barrier produced similar results correlating with our barrier integrity studies performed using EVOM meter. Since we observed a striking difference in TEER values between brain endothelium treated with cytokines at day3, 3 d time point was chosen to check the barrier solute permeability. While no difference between control and IL-1β treated brain endothelial FITC-dextran extravasation/permeability was observed, both TNF-α and IFN-γ strikingly decreased solute permeability at all times starting from 30 min to 6 h compared to untreated controls (Figure 1b). This experiment accurately correlates the barrier integrity with solute permeability and helped to rely more the barrier integrity measurements in our further experiments using EVOM meter for longer time points.

### 1c. Effect of mouse cytokines on endothelial + astrocyte co-culture barrier studies (Contact dependent co-cultures)

#### Control

Under untreated conditions, the TEER resistance of brain endothelial cells gradually decreased from day 1 (106 ± 0.5% to that of t = 0 (baseline)) through day 7 (to 65.9 ± 1.4% of baseline). At day 0 the resistance of untreated co-cultures was 208.33 ± 4.05 ohms/cm^2 ^and at day7 resistance was 128.67 ± 3.38 ohms/cm^2^.

#### TNF-α

TNF-α significantly increased TEER of brain endothelium until day 3, after which barrier decreased, (TEER values remained higher than control (Figure 1)). TEER peaked at day 3 (119 ± 1.4% of baseline). At day 7 the resistance of TNF-α treated brain endothelium remained higher than controls (92.1 ± 2.4 vs. 65.9 ± 1.4%). At day 0 the resistance of TNF-α treated co-cultures was 212.67 ± 4.17 ohms/cm^2 ^and at day 7, resistance was 197.67 ± 6.1 ohms/cm^2^.

#### IL-1β

IL-1β also significantly increased TEER until day 2, after which barrier gradually decreased. The resistance of IL-1β treated cells was maximal at day 1 (124.3 ± 5.3% of baseline). At day 7 the resistance of IL-1β treated endothelium was only slightly higher than controls (71.9 ± 6.5 vs. 65.9 ± 1.4%). At day 0, resistance of IL-1β treated co-cultures was 215.67 ± 2.66 ohms/cm^2 ^and at day7, resistance was 148.67 ± 16.37 ohms/cm^2^.

#### IFN-γ

The fractional increase in the TEER of brain endothelium treated with IFN-γ was greater than that of other 2 cytokines at all time points. The resistance of brain endothelium treated with IFN-γ was maximal level at day 5 (167.2 ± 4.7% of baseline). The resistance decreased from day 5, but remained higher than untreated brain endothelium (113 ± 16 vs. 65.9 ± 1.4%) (Figure 1c). At day 0 the resistance of IFN-γ treated co-cultures was 202 ± 2.08 ohms/cm^2 ^and at day7 resistance was 237.67 ± 38.28 ohms/cm^2^. The rank order of TEER in this experimental model was IFN-γ>TNF-α>IL-1β>Con. Inset shows the mode of culture and treatment.

### 1d Effect of mouse cytokine exposure on endothelial + astrocyte co-culture barrier studies (Contact independent co-culture)

#### Control

Endothelial cells cultured with astrocytes in a contact-independent model showed a similar response to that of the cells in a contact-dependent model with minor exceptions. Control TEER significantly increased at day 1, and was the time of maximal resistance (to 125.6 ± 2.4% of that at baseline), differing with the resistance of cells in contact-dependent studies. The resistance gradually decreased till day 7 (to 65.1 ± 2.6% of baseline TEER). At day 0 the resistance of untreated co-cultures was 188 ± 7.2 ohms/cm^2 ^and at day 7, resistance was 124 ± 3.5 ohms/cm^2^.

#### TNF-α

TNF-α treated brain endothelium significantly increased TEER at day 1 which gradually decreased at later time points. TEER peaked at day 1 (123.5 ± 1.6% of baseline). At day 7, the resistance of TNF-α treated cells remained higher than that of untreated control endothelium (75.87 ± 0.4% vs. 65.1 ± 2.6%). At day 0 the resistance of TNF-α treated co-cultures was 180.33 ± 8.37 ohms/cm^2 ^and at day 7, resistance was 161 ± 10.0 ohms/cm^2^.

#### IL-1β

IL-1β increased the resistance of brain endothelial cells at day 1 followed by a significant decrease in the resistance at day 7. The resistance was maximal at day 1 (131.2 ± 1.1% of baseline). The resistance of brain endothelial cells treated with IL-1β was similar to that of untreated brain endothelial cells at day 7 (65.32 ± 3.7% vs. 65.15 ± 2.6%). At day 0 the resistance of IL-1β treated co-cultures is 156 ± 8 ohms/cm^2 ^and at day7 resistance is 125.33 ± 0.8 ohms/cm^2^.

#### IFN-γ

IFN significantly increased the TEER of brain endothelial cells starting at day 1 through day 7. The maximal resistance was observed at day 2 (154.7 ± 2.6% over baseline, data not shown). Interestingly, the resistance of brain endothelial cells treated with IFN-γ remained higher than that of other cytokines or controls at day 7: 105.6 ± 9% (IFN-γ) > 75.87 ± 0.4% (TNF-α) > 65.15 ± 2.6% (control) = 65.32 ± 3.7% (IL-1β) (Figure 1d). The rank order of TEER in this experimental model was IFN-γ>TNF-α>IL-1β≈Con. Inset shows the mode of culture and treatment. At day 0 the resistance of IFN-γ treated co-cultures was 156.67 ± 8.17 ohms/cm^2 ^and at day 7, resistance was 313.33 ± 1.45 ohms/cm^2^.

### Figure 2. Effect of human cytokines on mouse brain endothelium + human astrocyte co-culture barrier studies

**Figure 2:**
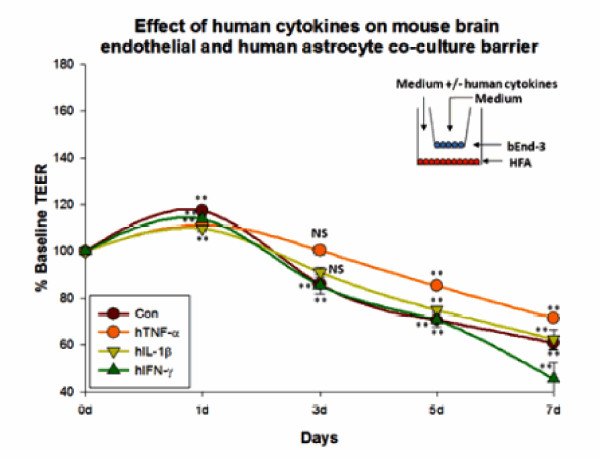
**Effect of human cytokines on human astrocytes in contact-independent mouse brain endothelial co-culture barrier**. Astrocytes were treated with human cytokines (TNF-α (20 ng/ml), IL-1β (20 ng/ml) and IFN-γ (1000 U/ml)) in a contact independent bEnd-3/HFA co-culture system. Resistance was recorded daily. hIFN-γ treated co-cultures from 5 d- 7 d showed decreased barrier compared to other treatment and control conditions. Inset shows the mode of co-culture system and cytokine addition. Bars indicate standard error. Repeated measures ANOVA with Dunnett's post-hoc test. *p < 0.05 was considered to be statistically significant, **p < 0.01 very significant, and ***p < 0.001 highly significant.

Treatment mode. Endothelial cells in the apical side (insert) were incubated in normal media, whereas astrocytes in the basal side were treated with media containing human cytokines.

#### Control

Endothelial cells co-cultured with astrocytes showed a progressive loss of TEER from days 3-7 (finally reaching 61.95 ± 1.6% of initial baseline). At day 0 the resistance of untreated co-cultures was 188.33 ± 0.8 ohms/cm^2 ^and at day 7, resistance was 116.67 ± 3.1 ohms/cm^2^.

#### TNF-α

We found that TNF-α treatment of astrocytes also decreased endothelial barrier resistance from days 3-7. Barrier resistance was almost similar to that of controls at day 7, but was greater than controls (71.51 ± 1.9 vs. 61.95 ± 1.6%). At day 0 the resistance of TNF-α treated co-cultures is 176.67 ± 1.4 ohms/cm^2 ^and at day 7, resistance was 126.33 ± 3.4 ohms/cm^2^.

#### IL-1β

When astrocytes were incubated in IL-1β, we observed a progressive drop in barrier from days 3-7 days. Resistance in IL-1β treated co-cultures at day 7 was similar to that of controls (63.51 ± .8 vs. 61.95 ± 1.6%). At day 0 the resistance of IL-1β treated co-cultures was 172.67 ± 1.2 ohms/cm^2 ^and at day 7, resistance was 109.67 ± 1.45 ohms/cm^2^.

#### IFN-γ

When astrocytes were incubated with human IFN-γ, a significant drop in barrier was observed over days 3-7. The resistance of IFN-γ treated co-cultures at day 7 was lesser than that of controls (46.47 ± 5.4 vs. 61.95 ± 1.6%) (Figure 2). At day 0 the resistance of IFN-γ treated co-cultures was 208 ± 2.03 ohms/cm^2 ^and at day 7, resistance was 96.66 ± 2.9 ohms/cm^2^. The rank order of TEER in this experiment was TNF-α>IL-1β≈Con>IFN-γ. These results show that cytokine effects, (IFN-γ in particular) on brain endothelial barrier is cell-specific and depends on astrocyte vs. endothelial exposures.

### 3) Effect of cytokines on mouse brain endothelial cell metabolism

**Figure 3:**
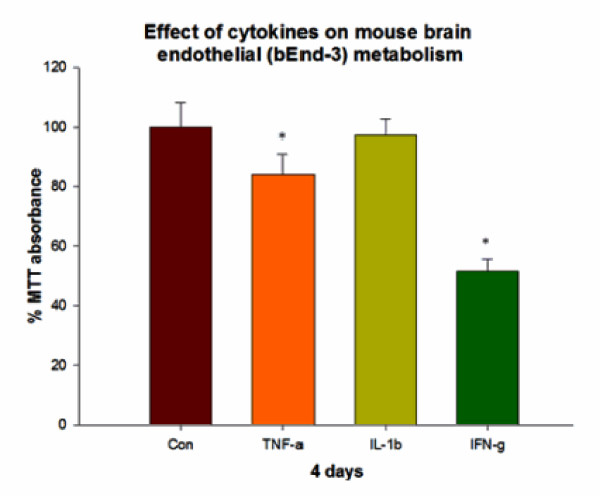
**Effect of mouse cytokines (TNF-α (20 ng/ml), IL-1β (20 ng/ml) and IFN-γ (1000 U/ml)) on mouse brain endothelial metabolism**. TNF-α (20 ng/ml) and IFN-γ (1000 U/ml)) significantly decreased mouse brain endothelial cell metabolism by 4 d but not IL-1β (20 ng/ml).

TNF-α at 4 d significantly decreased mouse brain endothelial metabolism (84.0 ± 6.9% baseline). IL-1β also slightly decreased cell metabolism of mouse brain endothelium but did not reach statistical significance (97.37 ± 5.2% baseline). IFN-γ showed a strong effect on mouse brain endothelial cells, decreasing metabolism more than the other 2 cytokines tested (reaching 51.5 ± 4% baseline) (Figure 3).

To further confirm our previous experiments using more physiologically relevant models, 2 separate human brain endothelial lines (HBMEC-3 and HCMEC-D3) and ECV-304 (an endothelial-like bladder carcinoma cell line) were studied in monoculture, as well as in co-culture with human astrocytes and barrier integrity investigated.

### 4a. Effect of human cytokine exposure on apical + basal sides of human brain endothelial (HCMEC-D3) mono-cultures

#### Control

Under untreated conditions, HCMEC-D3 barrier showed a progressive loss through day 7 (to 76.3 ± 1.0% of baseline). At day 0 the resistance of untreated mono-cultures was 297.33 ± 5.04 ohms/cm^2 ^and at day 7, resistance was 233.33 ± 2.66 ohms/cm^2^.

#### TNF-α

A prominent decrease in HCMEC-D3 barrier treated with TNF-α was observed. At day 7 the barrier integrity was considerably lower than that of controls (50.13 ± 0.6 vs. 76.3 ± 1.0% of baseline). At day 0 the resistance of TNF-α treated cultures was 297.33 ± 3.71 ohms/cm^2 ^and at day 7, resistance was 166 ± 1.73 ohms/cm^2^.

#### IL-1β

A gradual decrease in the HCMEC-D3 barrier treated with IL-1β was also observed until day 7. However, the barrier of HCMEC-D3 treated with IL-1β was similar to that of controls. At day 7 the barrier of IL-1β treated HCMEC-D3 was same to that of controls (73.07 ± 0.3 vs. 76.3 ± 1.0% of baseline). At day 0 the resistance of IL-1β treated cultures was 298.67 ± 1.73 ohms/cm^2 ^and at day 7, resistance was 226 ± 1.0 ohms/cm^2^.

#### IFNγ

**Figure 4:**
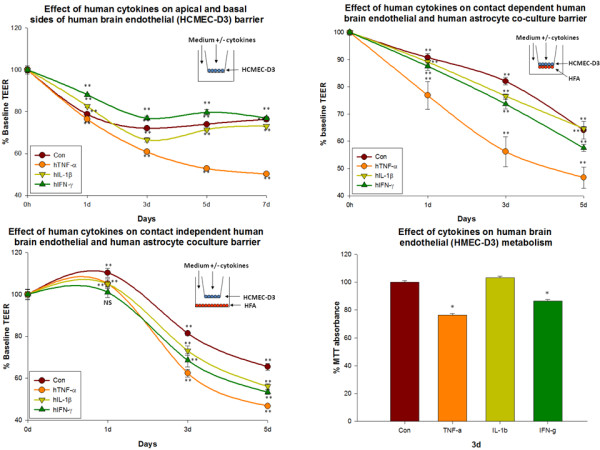
**Effect of human cytokines on HCMEC-D3 mono-culture barrier and HCMEC-D3/HFA co-culture barrier**. a) Effect of human cytokines (TNF-α (20 ng/ml), IL-1β (20 ng/ml) and IFN-γ (1000 U/ml)) applied to apical + basal sides of human brain endothelial (HCMEC-D3) mono-cultures. Resistance was recorded daily. Significant increase in the resistance of human brain endothelium treated with cytokines in a rank order of IFN-γ ≈ Con ≈ IL-1β > TNF-α was observed. Inset shows the mode of culture and cytokine treatment. Bars indicate standard error. Repeated measured ANOVA with Dunnett's post-hoc test. *p < 0.05 was considered to be statistically significant, **p < 0.01 very significant, and ***p < 0.001 highly significant. b) Effect of human cytokines (TNF-α (20 ng/ml), IL-1β (20 ng/ml) and IFN-γ (1000 U/ml)) on HCMEC-D3/HFA contact dependent co-culture barrier. Human cytokines were added to both apical and basal sides of the contact dependent co-culture system and TEER recorded daily. Co-cultures treated with TNF-α showed a higher loss in barrier integrity than other conditions. The rank order of this experiment is Con≈IL-1β>IFN-γ> TNF-α *p < 0.05 was considered to be statistically significant, **p < 0.01 very significant, and ***p < 0.001 highly significant. c) Effect of human cytokines (TNF-α (20 ng/ml), IL-1β (20 ng/ml) and IFN-γ (1000U/ml)) on HCMEC-D3/HFA contact independent co-culture barrier. Human cytokines were added to both apical and basal chamber of the co-culture system and TEER recorded daily. Co-cultures treated with cytokines showed lesser barrier integrity than untreated controls. The rank order of this experiment is Con> IL-1β ≈IFN-γ > TNF-α *p < 0.05 was considered to be statistically significant, **p < 0.01 very significant, and ***p < 0.001 highly significant. d) Effect of human cytokines (TNF-α (20ng/ml), IL-1β (20ng/ml) and IFN-γ (1000U/ml)) on HCMEC-D3 metabolism. TNF-α (20ng/ml) and IFN-γ (1000U/ml)) significantly decreased mouse brain endothelial cell metabolism by 3 d but not IL-1β (20ng/ml).

The percentage increase in IFN-γ treated HCMEC-D3 was slightly greater than that of other 2 cytokines at all time points. The resistance of IFN-γ treated cultures at day 7 was same as that of controls (76.87 ± 0.7 vs. 76.3 ± 1.0%) (Figure 4a). At day 0 the resistance of IFN-γ treated cultures is 289.33 ± 3.33 ohms/cm^2 ^and at day 7, resistance was 228.67 ± 1.850 ohms/cm^2^. The rank order of TEER in this experimental model was IFN-γ>IL-1β≈Con>TNF-α.

### 4b. Effect of human cytokines on human brain endothelial (HCMEC-D3) and human astrocyte contact dependent co-culture barrier

#### Control

Under control conditions contact dependent HCMEC-D3/HFA co-cultures' (incubated in 10% EBM2 in the apical side and 10% DMEM in the basal side) barrier showed a progressive loss till 5 d. At 5 d the barrier was 64.08 ± 3.2%. Resistance of contact dependent co-cultures' barrier at day 0 was 176.33 ± 0.3 and at 5 d resistance was 113 ± 5.7 ohms/cm^2^)

#### TNF-α

TNF-α treated contact dependent co-culture barrier showed a striking loss in the barrier starting from 1 d till 5 d. The barrier was 46.68 ± 3.9% baseline. The resistance of TNF-α treated co-cultures barrier was 175.67 ± 1.33 ohms/cm^2 ^and at 5 d the resistance was 82 ± 7.0 ohms/cm^2^.

#### IL-1β

IL-1β treated co-cultures barrier was slightly lower but almost similar to that of control co-cultures barrier. At 5 d the barrier was 64.67 ± 0.7% of baseline. The resistance values of IL-1β treated co-cultures at day 0 was 189.67 ± 1.2 ohms/cm^2 ^and at 5 d the resistance was 122.67 ± 1.45 ohms/cm2

#### IFN-γ

IFN-γ treated co-cultures barrier was lower compared to control co-cultures barrier. At 5 d the barrier was 57.58 ± 1.3% of baseline. Resistance of IFN-γ treated co-cultures barrier at 0 d was 187 ± 2.0 ohms/cm^2 ^and at 5 d resistance was 107.6 ± 2.6 ohms/cm^2 ^(Figure 4b). The rank order of TEER in this experimental model was Con≈IL-1β>IFN-γ>TNF-α.

### 4c. Effect of human cytokine exposure on human brain endothelial (HCMECD-3) and human astrocyte contact-independent co-culture barrier

#### Control

Under control conditions, HCMEC-D3/HFA contact independent co-cultures barrier showed a slight increase day1 followed by a gradual decrease. At day 5 the barrier of the co-culture was (to 65.46 ± 1.6% of baseline). At day 0 the resistance of untreated co-cultures was 164.33 ± 1.45 ohms/cm^2 ^and at day 5, resistance was 119.67 ± 2.1 ohms/cm^2^. The barrier was completely lost after 5 d.

#### TNF-α

A prominent decrease in HCMEC-D3/HFA co-culture barrier treated with TNF-α was observed. At day 5 the barrier integrity was considerably lower than that of controls (46.75 ± 0.6 vs. 65.46 ± 1.6% of baseline). At day 0 the resistance of TNF-α treated co-cultures was 173.33 ± 1.85 ohms/cm^2 ^and at day 5, resistance was 99.66 ± 0.8 ohms/cm^2^. The barrier was completely lost after 5 d.

#### IL-1β

A gradual decrease in the HCMEC-D3/HFA co-culture barrier treated with IL-1β was also observed from day1 until day 5. At day 5 the barrier of IL-1β treated HCMEC-D3 was slightly less than that of untreated co-cultures (56.08 ± 1.3 vs. 65.46 ± 1.6% of baseline). At day 0 the resistance of IL-1β treated co-cultures was 169.33 ± 3.1 ohms/cm^2 ^and at day 5, resistance was 110.33 ± 1.76 ohms/cm^2^.

#### IFN-γ

A gradual decrease in the IFN-γ treated HCMEC-D3/HFA co-cultures was observed. The resistance of IFN-γ treated cultures at day 5 is lesser than controls (53.37 ± 1.0 vs. 65.46 ± 1.6%) (Figure 4c). At day 0 the resistance of IFN-γ treated co-cultures is 168.67 ± 3.3 ohms/cm^2 ^and at day 5, resistance is 106.33 ± 1.45 ohms/cm^2^. The rank order of TEER in this experimental model was Con>IL-1β≈IFN-γ>TNF-α.

### 4c. Effect of cytokines on human brain endothelium (HCMEC-D3) metabolism

TNF-α at 3 d significantly decreased cell metabolism of HCMEC-D3 (76.49 ± 1.1% baseline control). IL-1β did not affect HCMEC-D3 cell metabolism (103.1 ± 1.1% baseline control). IFN-γ also significantly decreased HCMEC-D3 brain endothelial cell metabolism (86.57 ± 0.9% baseline control) (Figure 4d).

### 5a. Effect of human cytokine exposure on apical + basal sides of human brain endothelial (HBMEC-3) mono-cultures

**Figure 5:**
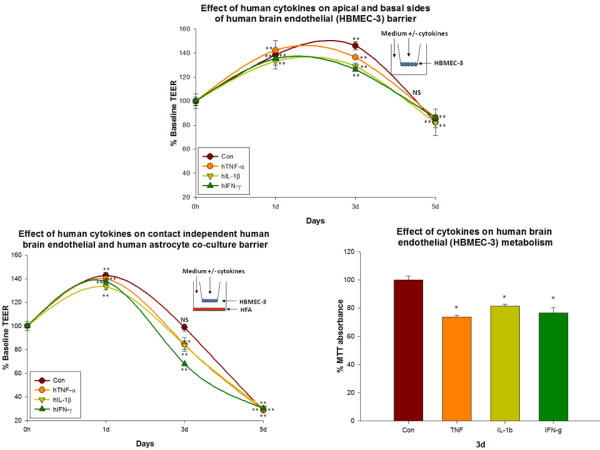
**Effect of human cytokines on HBMEC-3 mono-culture barrier and HBMEC-3/HFA co-culture barrier**. a) Effect of human cytokines (TNF-α (20 ng/ml), IL-1β (20 ng/ml) and IFN-γ (1000 U/ml)) applied to apical + basal sides of human brain endothelial (HBMEC-3) mono-cultures. Resistance was recorded daily. No significant difference in the resistance of cytokine treated HBMEC-3 barrier to that of untreated HBMEC-3 barrier was noted in this experiment. Bars indicate standard error. Repeated measured ANOVA with Dunnett's post-hoc test. *p < 0.05 was considered to be statistically significant, **p < 0.01 very significant, and ***p < 0.001 highly significant. b) Effect of human cytokines (TNF-α (20 ng/ml), IL-1β (20 ng/ml) and IFN-γ (1000 U/ml)) on HBMEC-3/HFA co-culture barrier. Human cytokines were added to both apical and basal sides of the co-culture system and TEER recorded daily. Co-cultures treated with cytokines showed slightly lesser barrier integrity than untreated controls. The rank order of this experiment is Con> IL-1β ≈TNF-α> IFN-γ *p < 0.05 was considered to be statistically significant, **p < 0.01 very significant, and ***p < 0.001 highly significant. c) Effect of human cytokines (TNF-α (20 ng/ml), IL-1β (20 ng/ml) and IFN-γ (1000 U/ml)) on HCMEC-D3 metabolism. TNF-α (20 ng/ml), IL-1β (20 ng/ml) and IFN-γ (1000 U/ml)) significantly decreased mouse brain endothelial cell metabolism by 3 d.

At confluence, HBMEC-3 cultures were treated with 10% RPMI with or without cytokines on both apical + basal sides. No significant effect of cytokines on HBMEC-3 barrier integrity was noted at any time point. The barrier integrity of cytokine treated cultures was similar to that of untreated cultures. However at day3 the barrier of the untreated cultures was slightly higher than that of other cytokine treated cultures. On day 5 barrier of the culture systems were the same (Con (82.39 ± 11.0% vs. baseline, resistance at 0 d = 245.33 ± 7.5 and at 5 d = 205.33 ± 2.85 ohms/cm^2^) vs. TNF-α (86.1 ± 2.3%, resistance at 0 d = 270 ± 7.6 and at 5 d = 234 ± 5.5 ohms/cm^2^) vs. IL-1β (81.87 ± 4.0%, resistance at 0 d = 267 ± 13.89 and at 5 d = 221.67 ± 9.1 ohms/cm^2^) vs. IFN-γ (86.1 ± 1.4%, resistance at 0 d = 260.67 ± 7.5 and at 5 d = 226 ± 3.2 ohms/cm^2^) (Figure 5a).

### 5b. Effect of human cytokine exposure on human brain endothelial (HBMEC-3) and human astrocyte contact independent co-culture barrier

#### Control

Under untreated conditions, HBMEC-3/HFA co-cultures barrier integrity was maintained until day 3 (98.71 ± 3.1% vs. baseline, resistance at 0 d = 232.67 ± 8.8 and at 3 d = 229.67 ± 7.3 ohms/cm^2^). On day 5 the barrier in co-culture decreased dramatically (28.65 ± 0.2% of baseline, resistance at 5 d = 66.67 ± .6 ohms/cm^2^).

#### TNF-α

TNF-α treated HBMEC3/HFA co-culture's barrier was similar to that of untreated co-cultures at day 1. However, by day 3 TNF-α treated co-culture barrier was reduced to less than that of controls (84.3 ± 5.8 vs. 98.71 ± 3.1%, resistance at 0 d = 230.67 ± 3.84, 3 d = 194.67 ± 13.3 ohms/cm^2^). By day 5, barrier was similar to controls (28.9 ± 0.5 vs. 28.65 ± 0.2%, resistance at 5 d = 66.66 ± 1.2 ohms/cm^2^).

#### IL-1β

Barrier in IL-1β treated HBMEC-3/HFA co-cultures followed the same pattern as TNF-α treated co-cultures. At day 3, IL-1β treated co-culture barrier was lower than that of controls (83.7 ± 3.6 vs. 98.71 ± 3.1%, resistance at 0 d = 219.67 ± 8.5 and at 3 d = 184 ± 8 ohms/cm^2^). At day 5 the barrier was dramatically reduced and was similar to that of controls (30.5 ± 0.2 vs. 28.6 ± 0.2%, resistance at 5 d = 67 ± 0.57 ohms/cm^2^).

#### IFN-γ

No significant difference in the barrier of IFN-γ treated HBMEC-3/HFA co-cultures was observed at day 1. However, on day3, IFN-γ treated co-culture barrier was lower than controls and other cytokine treated HBMEC-3/HFA co-cultures (67.7% vs. 98.71 ± 3.1%, resistance at 0 d = 215.67 ± 3.4 and at 3 d = 146 ohms/cm^2^). At day5 the barrier was similar to controls (and other cytokine treated HBMEC-3/HFA co-cultures) (30.29 ± 0.3 vs. 28.65 ± 0.2%, resistance at 5 d = 65.33 ± 0.6 ohms/cm^2^) (Figure 5b).

### 5c. Effect of cytokines on HBMEC-3 metabolism

TNF-α, IL-1β and IFN-γ significantly decreased HBMEC-3 brain endothelial metabolism by day3. While TNF-α decreased HBMEC-3 metabolism to 73.71 ± 1.4% of control levels, IL-1β decreased HBMEC-3 metabolism to 81.44 ± 1.4% and IFN-γ to 76.64 ± 3.6% of control levels (Figure 5c).

### 6a. Effect of human cytokine exposure on apical + basal sides of ECV-304 mono-cultures

#### Control

Under control conditions, a progressive loss of barrier was observed in ECV-304 monolayers through day 7 (to 47.8 ± 1.2% of baseline). At day 0 the resistance of untreated cultures was 353.67 ± 3.33 ohms/cm^2 ^and at day 7, resistance was 181.33 ± 2.9 ohms/cm^2^.

#### TNF-α

A slight decrease in the ECV-304 barrier treated with TNF-α was observed until day 7. However, at day 7 the barrier was still higher than controls (81.72 ± 1.6 vs. 47.8 ± 1.2% of baseline). At day 0 the resistance of TNF-α treated cultures is 367.67 ± 3.5 ohms/cm^2 ^and at day 7, resistance is 287.33 ± 12.7 ohms/cm^2^).

#### IL-1β

A gradual decrease in the barrier formed by ECV-304 treated with IL-1β was also observed until day 7. However, at day 7 the barrier was still slightly higher than controls (60.3 ± 2.2 vs. 47.8 ± 1.2% of baseline). At day 0 the resistance of IL-1β treated cultures is 357.67 ± 2.4 ohms/cm^2 ^and at day 7, resistance is 240 ± 12.6 ohms/cm^2^).

#### IFN-γ

**Figure 6:**
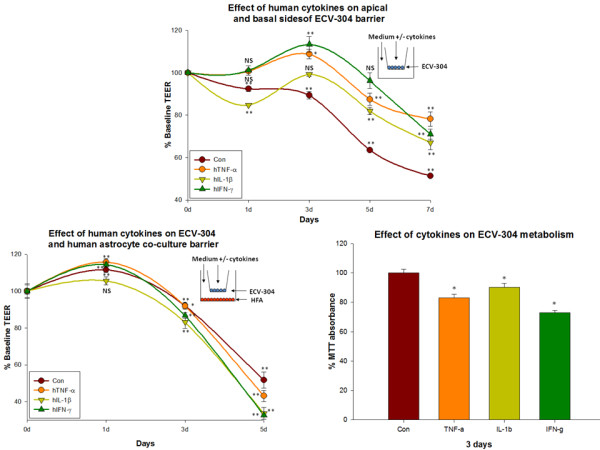
**Effect of human cytokines in ECV-304 mono-culture barrier and ECV-304/HFA co-culture barrier**. a) Effect of human cytokines (TNF-α (20 ng/ml), IL-1β (20 ng/ml) and IFN-γ (1000 U/ml)) applied to apical + basal sides of ECV-304 mono-cultures. Resistance was recorded daily. Significant increase in the resistance of human brain endothelium treated with cytokines in a rank order of IFN-γ ≈ TNF-α ≈> IL-1β > Con was observed. Inset shows the mode of culture and cytokine treatment. Bars indicate standard error. Repeated measured ANOVA with Dunnett's post-hoc test. *p < 0.05 was considered to be statistically significant, **p < 0.01 very significant, and ***p < 0.001 highly significant. b) Effect of human cytokines (TNF-α (20 ng/ml), IL-1β (20 ng/ml) and IFN-γ (1000 U/ml)) on contact independent ECV-304/HFA co-culture system. Resistance was recorded daily. After 5 d barrier was pronouncedly lost in all conditions. TEER readings obtained until 5 d were plotted to observe the effect of human cytokines on species matched co-culture barrier. A rank order of Con>TNF-α>IL-1β ≈ IFN-γ was observed. Inset shows the mode of contact dependent system used and cytokine addition. Bars indicate standard error. Repeated measures ANOVA with Dunnett's post-hoc test. *p < 0.05 was considered to be statistically significant, **p < 0.01 very significant, and ***p < 0.001 highly significant. c) Effect of human cytokines (TNF-α (20 ng/ml), IL-1β (20 ng/ml) and IFN-γ (1000 U/ml)) on in vitro cell metabolism. TNF-α (20 ng/ml), IL-1β (20 ng/ml) and IFN-γ (1000 U/ml)) significantly decreased ECV-304 metabolism by 3 d. Bars indicate standard error. One way ANOVA with Dunnett's post-test. *p < 0.05 was considered to be statistically significant, **p < 0.01 very significant, and ***p < 0.001 highly significant.

The fractional increase in ECV-304 barrier treated with IFN-γ was greater than that of other 2 cytokines at all time points. The resistance of ECV-304 treated with IFN-γ was maximal level at day 3 (133.5 ± 2.1% of baseline, resistance at 0 d = 366 ± 2.08 and at 3 d = 415 ± 13.2 ohms/cm^2^). The resistance decreased from day 3, but still remained higher than that of untreated ECV-304 at day 7 (96.0 ± 2 vs. 47.8 ± 1.2%, resistance at 7 d = 260 ± 9.07 ohms/cm^2^) (Figure 6a). The rank order of TEER in this experimental model was IFN-γ>TNF-α>IL-1β>Con.

### 6b. Effect of human cytokine exposure on ECV-304 and human astrocyte contact independent co-culture barrier

#### Control

Compared to untreated ECV-304 mono-cultures, ECV-304/HFA co-cultures lost the barrier more rapidly and were almost equal to baseline by 7 d. The barrier at 5 d was 51.7 ± 4.3% of baseline. At day 0 the resistance of untreated co-cultures was 327.67 ± 13.2 ohms/cm^2 ^and at day 5, resistance was 169.67 ± 14.1 ohms/cm^2^.

#### TNF-α

ECV-304/HFA co-cultures treated with TNF-α also lost the barrier but remained higher than untreated co-cultures. At 5 d the barrier of TNF-α treated co-culture was lower than controls (43.13 ± 3.1 vs. 51.7 ± 4.3% of baseline). At day 0 the resistance of TNF-α treated co-cultures is 327.67 ± 3.8 ohms/cm^2 ^and at day 5, resistance is 141.33 ± 10.3 ohms/cm^2^.

#### IL-1β

A rapid decrease in the ECV-304/HFA co-culture barrier treated with IL-1β was also observed until 5 d. At 5 d the barrier was still lower than controls (33.73 ± 3.3 vs. 51.7 ± 4.3% of baseline). At day 0 the resistance of IL-1β treated co-cultures is 335.67 ± 12.33 ohms/cm^2 ^and at day 5, resistance is 112.67 ± 11.26 ohms/cm^2^.

#### IFN-γ

IFN-γ treated co-cultures lost the barrier in a similar fashion to IL-1β treatment. At 5 d the barrier of co-cultures treated with IFN-γ was lesser than untreated co-cultures (32.73 ± 0.3% vs. 51.7 ± 4.3% of baseline). At day 0 the resistance of IFN-γ treated co-cultures is 311.67 ± 4.9 ohms/cm^2 ^and at day 5, resistance is 102.67 ± 1.0 ohms/cm^2 ^(Figure 6b). The rank order of TEER in this experimental model was Con>TNF-α>IL-1β≈IFN-γ. After 5 d both untreated and treated co-cultures' barrier was almost close to the baseline, indicating that more than the effect of cytokines, species matched stressed astrocytes can induce a more potent barrier permeability.

### 6c. Effect of cytokines on ECV-304 metabolism

All 3 cytokines in used in the study decreased ECV-304 metabolism. While TNF-α decreased ECV-304 metabolism to 83.13 ± 2.5% to baseline control, IL-1β decreased ECV-304 metabolism to 90.26 ± 2.5% and IFN-γ to 72.8 ± 1.7% (Figure 6c).

## Discussion

The neurovascular unit is a highly organized functional complex composed of neurons, their associated glia and microvessels which match cerebral blood flow with metabolism [19,62-64]. This unit is further divided into gliovascular units in which astrocytes support the function of neurons and communicate with the associated microvasculature. Astrocytes play a central role in integrating this functional unit. These neuro- and gliovascular units sense changes in local metabolism and synchronize functions between the involved cell types during normal physiological regulation [62,65]. However, during pathological conditions the cumulative influences of several internal and external factors may significantly alter this balance, to compromise the normal BBB. Dysregulation of the BBB appears to be a critical step in the pathogenesis of many CNS disturbances. Severely compromised BBB function is observed in many clinical conditions including brain trauma, ischemic stroke, meningitis, glioma, Alzheimer's disease and multiple sclerosis [3-10]. Such disruptions in the BBB play a pivotal role in aggravating many forms of cerebrovascular pathology by intensifying inflammatory responses within the CNS environment [66].

IFN-γ has been reported to decrease endothelial barrier [52,67-69], however it is worth noting that most of these studies have been performed in non-CNS endothelial cells. Brain endothelial cells differ from other endothelial cells in many respects including highly organized tight junctions which restrict paracellular transport and depend on biochemical support and interaction with astrocytes and neurons [70,71]. We attempted to identify specific responses involving interactions between astrocytes, individual cytokines, individually and in combination, to isolate possible mediators of barrier dysregulation in cell- and cytokine-mediated pathological conditions. Interestingly, our present study found unique brain endothelial responses to astrocytes and cytokines (compared to other endothelial types). Treatment with cytokines (i.e. TNF-α, IFN-γ, IL-1β) did not reduce barrier, compared to controls and paradoxically, TNF-α (on mouse brain endothelium) and IFN-γ somewhat enhanced barrier in mono-culture conditions. The effect of these cytokines on brain endothelial barrier (also on ECV-304) persisted for 7 days. These results differ from some, (but not all) previous reports, and may reflect complex, cell- and species-specific interactions.

For instance, Wong et al., observed decreased electrical resistance in human 1° endothelial cultures after treatment with 500 U/ml of IFN-γ [69]. We also observed a similar decrease in barrier when astrocytes (but not endothelial cells or ECV-304 alone) were treated with IFN-γ (in co-culture). Importantly, the observed barrier tightening effect of IFN-γ was eliminated and reversed when astrocytes were treated with IFN-γ in co-culture. This clearly shows that factors released by astrocytes exposed to IFN-γ (but perhaps not IFN-γ directly on endothelial cells) may trigger endothelial signaling and barrier breakdown. This finding indicates that negative barrier effects of IFN-γ on endothelial cells may be indirect, and reflect the production of factors produced by the astrocytes in our study. Stressed astrocytes may secrete several classes of factors, acting on brain endothelial cells (and other barrier forming cells, e.g. ECV-304) to compromise barrier. Activated astrocytes are known to release several factors like MMPs, that are involved in barrier breakdown [72-74]. Clear differences in the effect of cytokines on barrier are seen in different sets of conditions in the present study. For example, while some reports suggest that IL-1β dysregulates barrier [75], we found that barrier was maintained in brain endothelial monolayers treated with IL-1β (not different from controls). Moreover, when both astrocytes and brain endothelial cells were treated with cytokines in co-culture, trans-cellular resistance of co-cultures treated with TNF-α or IFN-γ were lower than controls indicating that astrocyte stimulation is required for barrier dysregulation rather than cytokines alone. Similar results were also found for ECV-304 cells. IFN-γ mediated barrier dysregulation involves a specific action on astrocytes rather than a direct effect on the brain endothelium (Figure 2, 4b, 5b and 6b). These results indicate that the specific actions of TNF-α in brain endothelial barrier dysregulation involves a synergy between endothelium, astrocytes and astrocyte-secreted factors and suggests that IFN-γ indirectly dysregulates barrier/permeability through activation of astrocytes.

To determine if TEER changes might parallel changes in cell energy metabolism, mitochondrial respiration was measured in both human and mouse brain endothelium upon exposure to cytokines for 4 days using MTT. In normal medium, brain endothelial cells were metabolically active and TNF-α and IFN-γ each significantly depressed metabolism of both mouse and human brain endothelial cells at days 3 and day 4 (significant change in metabolism vs. controls). These results indicate that the increase in barrier seen in human and mouse cells does not reflect metabolic depression. Moreover, it is possible that this decreased endothelial cell metabolism might be an adaptive response against cytokines which protects the barrier by conserving energy and preventing cell border contraction. This effect seems more prominent in IFN-γ treated brain endothelium. Therefore, IFN-γ may either modifies extracellular matrix (ECM) composition or alters endothelial junctions to prevent barrier dysregulation, a phenomenon which deserves further study [76]. Importantly, while some prior reports indicate that IFN-γ injures cells during cerebral ischemia, recent reports also indicate that IFN-γ protects neurons from CD8 T cell mediated injury [77-79]. Moreover, microglia treated with IFN-γ and transplanted *in vivo *protect neurons by secreting neurotrophic factors [80]. In the same context, the observed beneficial barrier tightening effect of IFN-γ may indicate another set of positive effects of IFN-γ in BBB modulation.

The barrier of brain endothelial cells (and ECV-304) was elevated by IFN-γ in all studies, except when astrocytes were treated with IFN-γ in co-culture with endothelial cells (and ECV-304). These results indicate that cytokines (e.g. IFN-γ) may initiate different barrier responses depending on the types of cells contacted, acute vs. chronic timing, and the cytokine involved. Several studies have tried to determine mechanisms through which astrocytes modulate endothelial barrier using contact-dependent and independent co-culture models. Taking into consideration that intimate contact with astrocytes might alter endothelial barrier; we studied the effect of cytokines in a contact-dependent transwell system. Interestingly, our results were similar in both models, and might reflect species-specific differences. Porcine endothelial and rat glial cells have been shown to be a useful system for contact-dependent BBB studies [26]. Porcine and rat cells might thus be able initiate modulating signals despite species differences, which human and mouse co-cultures may not duplicate. Therefore, to match the species specificity both human brain endothelial monocultures (HCMEC-D3, HBMEC-3) as well as ECV-304, and human brain endothelial: human astrocyte co-cultures (HCMEC-D3/HFA, HBMEC-3/HFA) and ECV-304/HFA) were prepared and evaluated for cytokine responses. Interestingly, similar responses were observed using mouse brain endothelial (bEnd-3) mono-cultures and mouse brain endothelial: human fetal astrocyte (bEnd-3/HFA) co-cultures. While TNF-α and IFN-γ induced barrier in mono-cultures, cytokine treated co-cultures showed a rapid reduction in barrier. However, a pronounced decrease in mouse, human brain endothelium and ECV-304 barrier was observed when starved human astrocytes were used in species-matched co-cultures; barrier was severely decreased by 5 d compared to controls. This indicates that stressed astrocytes strongly promote barrier breakdown which may be further aggravated by elevated cytokine levels, rather than through direct effects of these cytokines on the endothelium.

Another important aspect of this study is the apparent resistance of endothelial cells to various stressful conditions. For example, although brain endothelium are quite resistant to external forces/factors, results with stressed astrocytes show that astrocytes can disturb endothelial barrier. During CNS disorders like ischemic stroke, stressed/activated astrocytes may increase production of cytokines/proteases and intensify other factors leading to BBB failure during CNS pathologies. Pro-inflammatory cytokines like TNF-α, IL-1α, IL-1β, IL-6, GM-GSF and chemokines like MCP-1 have been implicated in several forms of BBB breakdown [11-15,17,18,66]. Further, cytokine mediated chemokine modulation (e.g. IL-1β driven MCP-1) has also been implicated in BBB breakdown [81,82]. These results indicate that cytokines indirectly affect other barrier modulators. A consistent observation of this study is that astrocytes mediate cytokine mediated BBB breakdown. The pathophysiology of many CNS disorders such as cerebral ischemia, MS, glioma and brain trauma are closely associated with increased production of cytokines in the brain. The production of these resulting cytokines can strongly activate astrocytes to release factors that dysregulate BBB. Despite a paradoxical tightening of barrier in response to IFN-γ and TNF-α, the loss of barrier due to the effect of cytokines on astrocytes indicates that these coordinated cytokine-astrocyte interactions closely regulate pathological breakdown of the BBB and are model-specific.

## Conclusions

Physiologically, astrocytes positively modulate brain endothelial barrier by stabilizing the solute barrier. Cytokines may exert detrimental effects on barrier in a differential and cell-specific model by astrocyte activation. Under appropriate pathological conditions activated astrocytes might dysregulate barrier biochemically by secreting factors that dysregulated or degrade BBB components. Interestingly, we found that astrocyte conditioned medium itself did improve barrier, and that cytokines either had no effect on, or increased barrier. Conversely, medium conditioned by astrocytes in the presence of these cytokines reduced barrier. Therefore cell-specific targets which neutralize effects of cytokines towards astrocytes or astrocyte-derived products towards endothelial cells may be beneficial in attenuating barrier dysregulation in several forms of neuroinflammation.

## Competing interests

The authors declare that they have no competing interests.

## Authors' contributions

GVC conceived and performed the majority of the experiments, analyzed data and wrote the study. WEC assisted in performing experiments and revising the manuscript. SW and MJ assisted with experiments and revisions. AEE provided HBMEC- 3 cells and helped in revisions of the study. POC, IAR, BW provided HCMEC-D3 cell lines and helped in revisions of the study. MJM assisted in interpretation and revision of the study. AM assisted in the interpretation and revision of the study. JSA helped conceive, analyze and interpret data and assisted in writing the manuscript. All authors read and approved the final manuscript.

## Authors' information

Ganta Vijay Chaitanya, PhD, Department of Molecular and Cellular Physiology, Louisiana State University Health Sciences Center-Shreveport, Louisiana-71130

Walter Cromer, PhD, Department of Cell Biology and Anatomy, Louisiana State University Health Sciences Center- Shreveport, Louisiana-71130

Shannon Wells, MPH, Department of Molecular and Cellular Physiology, Louisiana State University Health Sciences Center-Shreveport, Louisiana-71130

Merilyn Jennings, BS, Department of Molecular and Cellular Physiology, Louisiana State University Health Sciences Center-Shreveport, Louisiana-71130

Anat Erdreich-Epstein, MD, Division of Hematology-Oncology, Departments of Pediatrics and Pathology, The Saban Research Institute at Children's Hospital Los Angeles and the Keck School of Medicine, University of Southern California, 4650 Sunset Boulevard, Mailstop#57, Los Angeles, California 90027.

P.O. Couraud, PhD, Department of Cell Biology, Université Paris Descartes, CNRS (UMR 8104), Paris, France, Inserm, U567,

Ignacio A. Romero, PhD, Department of Biological Sciences, The Open University, Milton Keynes, UK, Department of Medicine

Babette Weksler, PhD, Weill Medical College, New York, NY USA.

J. Michael Mathis, PhD, Department of Cell Biology and Anatomy, Louisiana State University Health Sciences Center-Shreveport, Louisiana-71130

Alireza Minagar, MD' Department of Neurology, Louisiana State University Health Sciences Center-Shreveport, Louisiana-71130,

J. Steven Alexander, PhD, Department of Molecular and Cellular Physiology, Louisiana State University Health Sciences Center-Shreveport, Louisiana-71130
